# Liver abscesses in dromedary camels: Pathological characteristics and aerobic bacterial aetiology

**Published:** 2014-12-12

**Authors:** M.A. Aljameel, M.O. Halima, A.E. ElTigani-Asil, A.S. Abdalla, M.M. Abdellatif

**Affiliations:** 1 *Department of pathology and diagnosis, Nyala Veterinary Research Laboratory, Nyala, Sudan*; 2 *Department of pathology and diagnosis, Veterinary Research Institute, Khartoum, Sudan*; 3 *Department of Veterinary Medicine, Faculty of Agriculture &Vet Med. Qassim University, Saudi Arabia*; 4 *Emergency Coordination Office, FAO, Nyala, South Darfur State, Sudan*; 5 *Department of Microbiology, Faculty of Science, Northern Borders University, Saudi Arabia*

**Keywords:** Aerobic bacterial, Dromedary camels, Liver abscesses, Pathology, Sudan

## Abstract

The study was carried out at Nyala abattoirs, South Darfur State, Sudan during a period from 2009 to 2011. Slaughtered camels (822) were examined for pathological changes of liver abscesses and identification of the involved aerobic bacteria. Grossly, a total of 111 (13.5%) liver abscesses were recorded in different camel ages; 90 (81.1%) were less than seven years old and 21 (18.9%) were more than seven years old. Histopathology of sectioned tissues revealed necrotic abscesses with infiltration of inflammatory cells, hydropic degeneration with swelling of hepatocytes comprising the sinusoid and different size of vacuoles in the hepatic cells. Proliferation of bile ducts with fibrous tissue and infiltration of inflammatory cells was also recorded. Investigation of bacteria revealed 90 aerobic isolates; they were identified to 52 (57.8%) gram positive cocci, 20 (22.2%) gram positive rods and 18 (20.0%) gram negative rods. *Staphylococcus* spp. (41.1%), *Corynebacterium* spp. (17.9%) and *Streptococcus* spp. (13.3%) were the most frequently identified bacteria involved in liver abscesses of camels in the region. Further studies are required to assess the pathogenicity of bacterial isolates from camel livers. This is particularly important from a public health perspective, since some people of Sudan are known to consume raw camel liver.

## Introduction

Camels are the most capable animal species in survival and production under harsh environmental conditions in marginal arid areas (Abbas and Tilley, 1990; Schwartz, 1992). It is well adapted to the climatic extremes and is well appreciated for its significance in the pastoral economy (Raziq and Younas, 2006).

Abscesses located in the internal organs are among the most prominent emerging problems of camels causing considerable losses in production and varying mortality rates (Bekele, 1999). Generally, they are only detected after the animals are slaughtered, because even hundreds of small abscesses or several large abscesses rarely cause clinical manifestation (Nasgaraja and Chengappa, 1998).

Liver abscesses in meat-producing animals represent a major factor that reduces the national income, either directly through condemnation of affected livers, or indirectly through effects on animal growth and production (Eid *et al.*, 1998).

Several studies have focused on bacterial pathogens as possible causative agents of abscesses in camels (Zubair *et al.*, 2004; Abubakar *et al.*, 2010; Stefan *et al.*, 2011; Priyanka and Kataria, 2012; Shiferaw *et al.*, 2012; Aljameel *et al.*, 2013a). *Staphylococci*, *Streptococci*, *Pseudomonas* spp., *Actinomyces* spp., *Corynebacterium pseudotuberculosis*, *Proteus* spp., *Enterobacter* spp., *Klebsiella pneumoniae*, *Bacillus* spp., and *Escherichia coli* were found to be the most frequent agents involved in liver abscesses (Hawari, 2008; Nourani and Salimi, 2013; Wael and Taha, 2013).

However in the Sudan, Wisal and Salim (2008) isolated *Actinomyces*, *Bacillus*, *Corynebacterium*, *Enterobacter*, *Escherichia*, *Pseudomonas*, *Salmonella* and *Staphylococcus* from camel contagious skin necrosis. Moreover, Aljameel *et al*. (2013a, b) isolated *Staphylococcus* spp., *Streptococcus* spp., *Crynebacterium pseudotuberculosis*, *Pseudomonas* ssp., *Klebsiella* spp. and *Actinomyces* (*Arcanobacterium*) *pyogenes* from lung, lymph nodes and spleenic abscesses of dromedary camels in South Darfur State, Sudan.

To our knowledge, no data were so far available concerning liver abscesses in dromedary camels in Sudan. Thus, this study was designed to investigate liver abscesses in camels in South Darfur State, Sudan.

## Materials and Methods

### Study district

The study was conducted from January 2009 to December 2011 at Nyala abattoirs, South Darfur State, Sudan.

### Sample collection

Camels of varying age and sex (822) were subjected to visual and physical examination for detection of liver abscesses. At post-mortem, liver abscesses were collected in duplicate. One sample was fixed in 10% buffered formal saline for histopathology while the other sample was placed in sterile containers, preserved in ice pack and transported to the Bacteriology Department at the Nyala Veterinary Research Laboratory for examination within two hours of collection.

### Pathological investigation

Gross pathology of liver was fully described and parts of formal saline fixed liver abscess (1cm^3^) were dehydrated in graded ethanol and embedded in paraffin wax. Sections (5 µm) were stained with haematoxylin and eosin according to the method described by Hewitson and Darby (2010).

### Bacterial cultivation and identification

The livers containing abscesses were flamed and a sharp incision was made in the lesion using a sterile blade under sterile conditions. Pus was collected with an inoculation loop and streaked on to plates containing 5% sheep defibrinated blood agar base (Scharlau, Ref. 01-352, Batch: 15518) and on MacConkey agar (Pronadisa, Cut. 1099.00), which were prepared according to manufacturer’s instructions (Bergery, 1984). The plates were incubated under aerobic and an- aerobic conditions at 37ºC for 24 - 48 h. The growing colonies were picked up with a sterile loop and sub-cultured for purification. Cultures were Gram-stained for assessment of morphology. Bacterial agents were identified by conventional biochemical characterization and API 20E system (API Analytical Profile Index, BioMerieu, France) (Quinn *et al.*, 2002; Barrow and Feltham, 1993).

## Results

### Prevalence of abscesses

Out of 822 examined camels, 111 (13.5%) exhibited liver abscesses. Of these, 70 (63.1%) were females and 41 (36.9%) were males. Ninety camels (81.1%) were less than seven years old and 21 (18.9%) were more than seven years old.

### Autopsy

Physical examinations of livers showed that the abscesses were encapsulated by a relatively thick layer of fibrous connective tissues capsule.

**Fig. 1:**
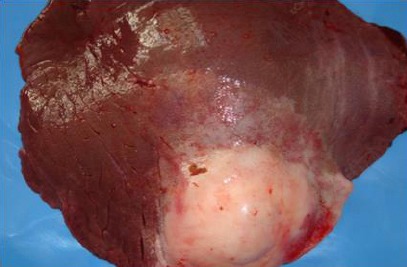
Gross appearance of a liver showing encapsulated abscess with a thick capsule.

**Fig. 2:**
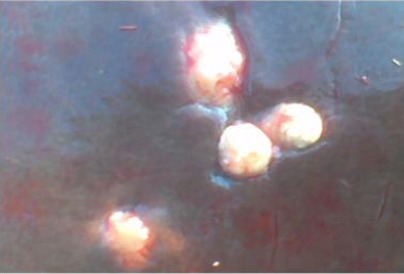
Gross appearance of surface of liver showing multiple calcified encapsulated abscesses.

**Fig. 3:**
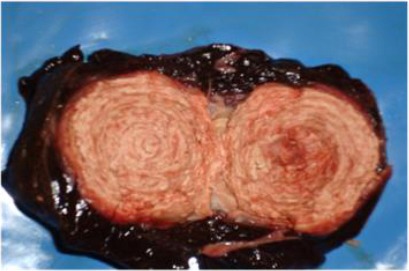
A cut section in liver showing onion shape abscess (*Corynebacterium pseudotuberculosis* was isolated).

**Fig. 4:**
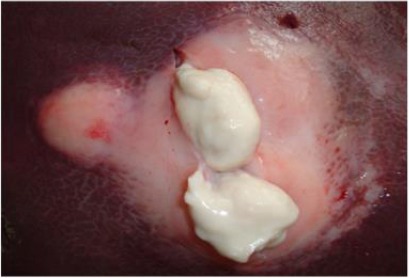
A cut section in a large liver abscess showing white creamy pus and a thick capsule.

Cross section of the necrotic areas revealed odorless thin, creamy or watery white, grey or yellowish pus in some lesions and areas of calcification in others (Figs. 1, 2, 3 and 4).

### Histopathology

**Fig. 5:**
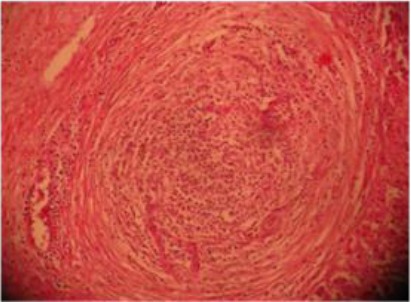
Histopathological section of liver abscess showing central necrotic area and infiltration of inflammatory cells surrounded by fibrous capsule. H&Ex40.

**Fig. 6:**
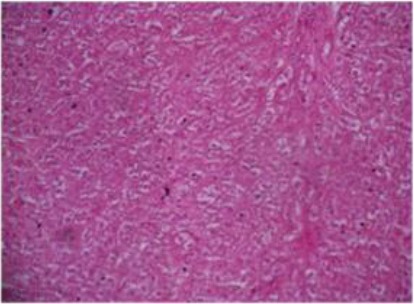
Histopathological section of liver abscess showing hydropic degeneration with swelling hepatocytes comprising the sinusoid. H&Ex40.

Liver sections revealed necrotic abscesses and infiltration of inflammatory cells (Fig. 5), and hydropic degeneration with swelling of hepatocytes comprising the sinusoid (Fig. 6).

**Fig. 7:**
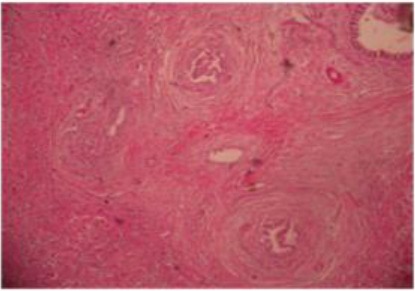
Histopathological section of liver abscess showing proliferation of bile duct with fibrous connective tissue and infiltration of inflammatory cells. H&Ex40.

**Fig. 8:**
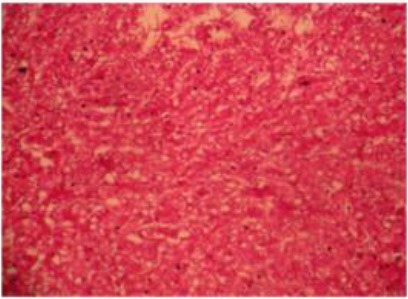
Histopathological section of liver abscess showing different size of vacuoles in the hepatic cells. H&Ex40.

Moreover, proliferation of bile ducts with fibrous tissue and infiltration of inflammatory cells (Fig. 7) and different size of vacuoles in the hepatic cells (Fig. 8).

### Bacteria isolated from liver abscesses

Ninety aerobic bacteria were isolated in this study, and found to belong to (11) genera (Table 1). Out of them, 52 (57.8%) were gram positive cocci (Table 2), while 20 (22.2%) were gram positive rods (Table 3) and 18 (20.0%) were gram negative rods (Table 4). *Staphylococcus* spp. (41.1%), *Corynebacterium* spp. (17.9%), and *Streptococcus* spp. (13.3%) were the most frequently identified genera involved in the liver abscesses of dromedary camels.

**Table 1:** Percentage of aerobic bacterial isolates from abscesses in liver of the dromedary camel in South Darfur State, Sudan.

Bacterial isolates	Percentage (%)
*Staphylococcus* spp.	37 (41.1 %)
*Streptococcus* spp.	12 (13.3 %)
*Corynebacterium* spp.	16 (17.9 %)
*Proteus* spp.	5 (5.6 %)
*Pseudomonas* spp.	3 (3.3 %)
*Arcanobacterium* spp.	2 (2.2 %)
*Escherichia* spp.	5 (5.6 %)
*Micrococcus* spp.	3 (3.3 %)
*Klebsiella* spp.	3 (3.3 %)
*Enterobacter* spp.	2 (2.2 %)
*Bacillus* spp.	2 (2.2 %)
Total	90 (100 %)

**Table 2:** Gram-positive cocci isolates from abscesses in liver of the dromedary camel in South Darfur State, Sudan.

No.	Bacterial isolates	Frequency	Percentage %
1	*Staphylococcus* ssp.		
	*S. aureus*	26	70.3 %
	*S. lentus*	3	8.1 %
	*S. hyicus*	2	5.4 %
	*S. caprae*	2	5.4 %
	*S. equorum*	2	5.4 %
	*S. hominis*	1	2.7 %
	*S. vitulus*	1	2.7 %
2	*Streptococcus* spp.		
	*Str. pyogenes*	7	58.3 %
	*Str. salivarius*	2	16.7 %
	*Str. uberis*	2	16.7 %
	*Str. agalactiae*	1	8.3 %
3	*Micrococcus* ssp.		
	*M. luteus*	2	66.7 %
	*M. varians*	1	33.3 %
	Total	52	57.8%

**Table 3:** Gram-positive rods isolated from abscesses in liver of the dromedary camel in South Darfur State, Sudan.

No.	Bacterial isolates	Frequency	Percentage %
1	*Corynebacterium* spp.		
	*C. pseudotuberculosis*	14	87.5 %
	*C. renali*	2	12.5 %
2	*Arcanobacterium* spp.		
	*A. pyogenes*	1	50.0 %
	*A. viscosus*	1	50.0 %
3	*Bacillus* spp.		
	*B. licheniformis*	2	100 %
	Total	20	22.2 %

**Table 4:** Gram-negative rods isolated from abscesses in liver of the dromedary camel in South Darfur State, Sudan.

No.	Bacterial isolates	Frequency	Percentage %
1	*Pseudomonas* spp.		
	*P. aeruginoas*	2	66.7 %
	*P. vesicularis*	1	33.3 %
2	*Proteus* spp.		
	*Pro. vulgaris*	3	60 %
	*Pro. mirabilis*	2	40 %
3	*Enterobacter* spp.		
	*En. aerogenes*	1	50 %
	*En. cloacae*	1	50 %
4	*Klebsiella* spp.		
	*K. pneumoniae ssp. pneumoniae*	1	33.3 %
	*K. pneumoniae ssp. ozaenae*	2	66.7 %
5	*Escherichia* spp.		
	*E. coli*	5	100 %
			

## Discussion

In the present study the prevalence of liver abscesses (13.5%) was slightly higher than those reported by previous investigators in Jordan (1.2%) (Al-Ani *et al.*, 1998) and in Iran (0.64%) (Nourani and Salimi, 2013). Our high prevalence rate could be attributed to environmental changes and husbandry practices, mixed herding, sharing of water and pasture, and migration with other animal species. Distribution of the pyogenic liver infections was found as 81.1% in camels less than seven years old and 18.9% in animals more than seven years old.

These findings suggested that the immune system of young camels is weaker than those adults, which make young camels more vulnerable to infection with pyogenic microorganisms (Devrajani *et al.*, 2010).

Histopathological sections showed necrotic areas and infiltration of inflammatory cells, hydropic degeneration with swelling of hepatocytes and vacuolation comprising the sinusoid, proliferation of bile ducts with fibrous tissue in the manner of granulomatous lesions signed chronic infection. Similarly Nourani and Salimi, (2013) noted the abundance of hepatocellular degeneration, necrosis and toxic hepatic lesions associated with liver abscesses of the dromedary camel.

The histological variations in size of liver abscesses observed in this study might suggest that the earliest lesion was a micro abscess, possibly induced by an embolus of bacteria in the hepatic sinusoid, followed by progression of the lesion to coagulative necrosis by involving adjacent hepatocytes and subsequently the lesion gradually changed into a pus-filled encapsulated mature abscess (Tehrani *et al.*, 2012).

Ninety aerobic bacteria, (57.8% gram positive cocci, 22.2% gram positive rods and 20% gram negative rods) were isolated and identified from liver abscesses considered in this study. This in agreement with Wael and Taha (2013) who isolated *Corynebacterium pseudotuberculosis*, *Escherichia coli* and *Staphylococcus* spp. from liver abscesses in dromedary camels. Moreover, this finding was consistent with previous reports showing that *Staphylococcus* spp., *Streptococcus* spp., *Pseudomonas aeruginosa* and *E. coli* are bacterial agents commonly isolated from lesions in liver of camels (Hawari, 2008; Abubakar *et al.*, 2010; Hegazy *et al.*, 2010).

In this study, *Corynebacterium pseudotuberculosis*, was isolated in a pure culture from a liver having an onion shape abscesses, a finding which is in agreement with Philippa and Jenifer (2012) who isolated *C. pseudotuberculosis* from liver abscesses in a mature alpaca. Such organism was isolated from an 11-year-old llama suffering from submandibular abscess (Lopes *et al.*, 2012). Importantly, the isolation of *Arcanobacterium pyogenes* in this study strongly suggested its involvement with liver abscesses. Based on available literature, *A. pyogenes* has not been previously incriminated as a pathogen causing abscesses of camels in the Sudan. Such organism enters the blood stream then disseminate to cause septic arthritis, suppurative lesions and abscesses in various organs and tissues, but mainly in the lungs and liver (Tolle *et al.*, 1989). In this study, we concluded that *Staphylococcus* spp., *Streptococcus* spp., and *Corynebacterium pseudotuberculosis* were the most incriminated bacteria in camels liver abscess.

Moreover, young camels are more sensitive to liver abscess bacteria. Therefore, further studies are needed to improve camel health and production by identifying other abscess-causing agents in camels and assessing the pathogenicity of bacterial isolates from camel liver. This is particularly important form a public health perspective, since some people of Sudan are known to consume raw camel liver.
